# Young Adults’ Understanding of Modifiable Risk Factors of Infertility

**DOI:** 10.1089/whr.2024.0058

**Published:** 2024-10-22

**Authors:** Lauren Lim, Meredith Hoppe, Lauren Kennedy, Allison Gunderson, Lingchen Wang, Neda Etezadi-Amoli

**Affiliations:** ^1^ Reno School of Medicine, University of Nevada, Reno, Nevada, USA.; ^2^ School of Public Health, University of Nevada, Reno, Reno, Nevada, USA.

**Keywords:** fertility, fertility awareness, infertility, lifestyle, medical education, lifestyle, preventative medicine, reproductive health

## Abstract

**Objective:**

Assess the knowledge of young adults regarding modifiable risk factors of infertility.

**Design:**

Web-based validated survey.

**Setting:**

University of Nevada, Reno (UNR).

**Subject(s):**

Undergraduate students at the UNR.

**Intervention(s):**

A survey encompassing participants’ demographics, understanding of infertility risk factors, willingness to modify behaviors to prevent infertility, personal significance of fertility, previous sources for fertility knowledge, and preferred sources for fertility education.

**Main Outcome Measure(s):**

Subject-reported knowledge of modifiable risk factors for infertility and value of fertility.

**Result(s):**

A total of 427 individuals responded. Thirty-seven percent of females indicated that oral contraceptive pills negatively impact their future fertility and 34.4% were unsure. Regarding prior use of long-acting reversible contraceptives on future fertility, 31.4% of females believed it had a negative impact and 36.9% were unsure of its impact. Only 21.7% of males thought testosterone had a negative impact on fertility. Participants were significantly more likely to avoid certain modifiable risk factors to prevent infertility than they were to avoid excessive alcohol to prevent liver disease (*p* = 0.008). The largest percentage of women reported obtaining the most information about their fertility from social media.

**Conclusion(s):**

Young adults would benefit from and are interested in a better understanding of their fertility and modifiable risk factors for infertility. Obstetrics and gynecology physicians and primary care providers can use these findings to guide education and address misconceptions.

## Introduction

Infertility is an emotionally and financially heavy burden for women and men, affecting up to one in six couples in Western countries.^1^ The American Society of Reproductive Medicine (ASRM) defines infertility as 12 months of unprotected and regular sexual intercourse without establishing a clinical pregnancy; the inability to achieve a pregnancy secondary to age, medical, sexual, reproductive history, physical findings, and diagnostic testing; or the need for medical intervention to achieve a pregnancy.^2^ The average parental age has increased in Western countries over recent decades, and this trend is expected to continue.^3^ Increased parental age can contribute to the increased need for assisted reproductive technology (ART) to establish pregnancy.^4–6^ Causes of infertility can be classified as nonmodifiable and modifiable. Nonmodifiable sources include abnormal sperm production and function**;** ovulation disorders; structural problems involving the uterus, cervix, or fallopian tubes; or primary ovarian insufficiency,^7–9^ while modifiable sources include body mass index (BMI), tobacco use, alcohol use, dietary factors, stress, exogenous testosterone, marijuana use, and sexually transmitted diseases (STDs).^10–14^

Young adults who wish to conceive in the future are often unaware of how lifestyle choices in early adulthood may influence one’s ability to conceive in the future.^15–17^ Knowledge gaps about fertility exist, as demonstrated by a study reporting the results of a fertility facts and risk factors quiz taken in 79 different countries.^15,16^ Furthermore, surveys collected in Denmark and Canada reported similar findings—many people who desire having children struggle on questionnaires evaluating their knowledge on fertility issues.^18,19^ This existing literature shows a disconnect between the value of fertility and the understanding of fertility.

Fertility education is shown to have a positive impact on reproductive outcomes due to better understanding of modifiable risk factors.^20,21^ Many studies address young adults’ knowledge on general fertility, likelihood of pregnancy, or ARTs.^16,18,19,21^ To our knowledge, few studies investigate undergraduate students’ perceptions on modifiable risk factors for infertility.^15,17,20^ Given the increased use of social media for health-related content, having up-to-date awareness of young adults’ understanding of fertility is critical.^22^ One study shows women aged 18–25 use social media daily for general health information and weekly for information relating to preconception or pregnancy.^23^ Current literature does not address whether young people are willing to change risky behaviors after learning about its impact on fertility. The primary objective of this study is to identify deficits in university students’ understanding of modifiable risk factors of infertility. The secondary objective is to understand how likely young adults are to make behavior changes to prevent infertility and to assess how they have previously learned about fertility.

## Materials and Methods

### Study design

The University of Nevada, Reno (UNR) Institutional Review Board determined this study exempt from full review. This study utilized a descriptive survey to analyze the understanding of young adults enrolled at the UNR between the ages of 18 and 26 on modifiable risk factors of infertility.

### Sample

#### Sample selection

UNR undergraduate and graduate students aged 18–26 were selected. Emails were dispatched to course instructors of Introduction to Biology, Cell Biology, Genetics, Introduction to Business, Introduction to Economics, Community Health Sciences, Psychology, Nutrition, and Human Development and Family Studies, as well as all fraternity and sorority leadership to gather responses from large courses in different majors. Emails included a flier outlining the purpose of the survey and requested permission for researchers to visit an in-person meeting or lecture to explain the survey and allow participants to complete the survey. Additionally, participants were recruited through fliers displayed across campus. No other campus staff, administration, or student groups disseminated the survey beyond allowing researchers to visit classes or club meetings to recruit participants. Students who were not enrolled at the UNR, younger than 18 years of age, or older than 26 years of age were excluded from the study. Students had to be enrolled in undergraduate courses at UNR.

#### Sample size

The sample size for this survey was determined using the Clopper–Pearson exact method with PASS 15 software.^24,25^ To ensure adequate precision, a proportion of 50% was assumed, which is the most conservative estimate for calculating the width of the confidence interval. This assumption guarantees that the calculated sample size provides a sufficient margin of error regardless of the actual observed proportion. Assuming a proportion of 50%, a sample size of 100 participants would result in a two-sided 95% confidence interval ranging from 39.8% to 60.2%, with a total width of 20.3% (±10.15%). Although the initial design aimed to collect a minimum of 100 participants to ensure basic precision in the results, there was no upper limit on the number of participants, which allowed us to achieve greater precision in our findings. Our survey ultimately collected a total of 427 participants. With this sample size, the 95% confidence interval for a proportion of 50% narrowed to 45.2% to 54.8%, yielding a confidence interval width of 9.7% (±4.85%).

#### Participants

Participants for the study were gathered on a volunteer basis. Based on responses, the participants in this study were from the courses Introduction to Biology, Cell Biology, Community Health Science, and Human Development and Family Studies as well as one sorority member and one fraternity member.

### Survey instrument

The web-based survey, consisting of 27 questions, was created using Qualtrics XM. The survey was designed to assess knowledge, attitudes, and perceptions regarding fertility and associated risk factors.

#### Survey development and validation

The survey was developed and validated in a series of steps.

1. Development: Survey questions were developed using information from the Centers for Disease Control and Prevention (CDC)^26,27^ and the ASRM,^14^ along with prior clinical experience and an extensive literature review using databases including but not limited to PubMed and Google Scholar.
2. Validation: The survey was validated in a two-step process:^28^
  • Expert review: Three board-certified obstetrics and gynecology (Ob/Gyn) physicians reviewed the survey to confirm its content addressed the study’s objectives.
  • Pilot testing: Ten graduate medical students completed the survey to evaluate the average completion time, question clarity, and interface usability. Feedback led to minimal adjustments in the survey.

Minimal adjustments were made to the survey in response to its validation. One survey question was added in regard to nonmodifiable risk factors for infertility, and the wording of “prior use” of contraceptives was clarified.

#### Survey structure

The survey questions (Supplementary Appendix SA1) comprised different sections designed to gather comprehensive data on the participants’ knowledge, attitudes, and perceptions related to fertility:

• Consent agreement: Before the survey began, participants were notified of the purpose of the survey and that it was voluntary and anonymous. They also were required to give consent for their participation before completing the survey.
• Demographics: Four questions gathered demographic information, including gender (participants’ chosen identity), sex (biological assignment at birth), age, and student status at the UNR.
• Knowledge assessment: Fifteen multiple-choice questions assessed knowledge of risk factors for infertility. Participants indicated whether a behavior positively impacted fertility, negatively impacted fertility, had no impact, or if they were unsure.
• Attitudes: Three Likert scale (1–5) questions evaluated the likelihood of participants changing behaviors that could negatively impact their health, such as risks associated with infertility, liver disease, and lung cancer.
• Subjective understanding: Three questions addressed participants’ subjective understanding of their fertility.
• Sources of learning: Two questions determined where participants had learned the most about fertility and where they wished they had learned about fertility.

#### Survey completion

During the validation process, the majority of participants taking the survey noted that completion of the survey took less than 5 minutes.

### Data collection

The web-based survey was created using Qualtrics XM. A QR code linked to the survey was generated and displayed on a projector during in-person meetings and on the flier posted across campus for participants to access the survey. A consent form that explained the purpose of the study and emphasized participation was voluntary and anonymous was displayed before the survey began. Researchers also explained this to participants during in-person meetings. Responses were collected between the months of February and April 2023.

### Statistics

The statistical analysis was conducted for three main sections: understanding of how different behaviors impact fertility, likelihood of changing behaviors, and attitudes regarding fertility awareness and importance.

#### Understanding of how different behaviors impact fertility

The variables in this section are nominal. Counts and percentages were used to describe these variables. To compare the responses between male and female participants for these nominal variables, chi-squared and Fisher’s exact tests were used. When the overall comparison indicated a significant difference, *post hoc* tests were performed to identify specific option differences.

#### Likelihood of changing behaviors

The variables in this section are ordinal. Means and standard deviations were used to describe these variables. Because the same individuals were compared regarding the likelihood of changing different behaviors, Friedman’s test for paired comparisons among the three questions was used. When the overall comparison showed a significant difference, pairwise comparisons were conducted. Bonferroni correction was used to adjust the *p* values of these pairwise tests to account for multiple comparisons.

#### Attitudes regarding fertility awareness and importance

The variables in this section are ordinal. Both means and standard deviations were used, as well as counts and percentages, to describe these variables. To compare responses between male and female participants for these ordinal variables, Wilcoxon rank-sum tests were used.

The significance level was set at 0.05 for all tests. All statistical analyses were performed using SPSS version 25.0.

## Results

### Demographics

The survey was completed by 427 UNR students. Most of the students were between 18 and 20 years of age (74.8% of men and 76.3% of women). There was no difference in age distribution between males and females (*p* = 0.332). Nearly 18% of participants were between 21 and 23 years of age, 2.8% between 24 and 26 years of age, and 3.5% were older than 26 years of age. Most participants were assigned female at birth and identified as women (73.5%, 71.7%).

### Understanding risk factors for fertility

The largest percentage of both females and males responded that stress, high blood pressure, an overweight or underweight BMI, and STDs negatively impact their fertility and that proper nutrition positively impacts fertility. However, when asked about the impact of the influenza vaccine, 37.5% of females and 46.1% of males were unsure. When questioned about the impact of hormones on fertility, about 50% of males were unsure if exogenous testosterone use affects fertility, and the majority of females were either unsure of the impact of prior use of oral contraceptives or believed it negatively impacted fertility. Similarly, the majority of females indicated that long-acting reversible contraceptions (LARCs) had either a negative impact on fertility or were unsure. Over half of both males and females responded that smoking tobacco and excessive alcohol intake negatively impact fertility. When asked about marijuana use and fertility, there was a discrepancy between males and females, with more females reporting a negative impact on fertility than males (*p* < 0.05). Table 1 illustrates the responses to questions regarding modifiable risk factors for infertility.

**Table 1.** Understanding of How Different Behaviors Impact Fertility

	Total (*n* = 427)	Male (*n* = 115)	Female (*n* = 312)	*p* value
How does stress impact your sex’s fertility?				0.012
Negatively impacts	322 (75.4%)	74 (64.3%)^a^	248 (79.5%)^a^	
Positively impacts	8 (1.9%)	4 (3.5%)	4 (1.3%)	
No impact	30 (7%)	12 (10.4%)	18 (5.8%)	
Unsure	67 (15.7%)	25 (21.7%)^a^	42 (13.5%)^a^	
How does obesity (BMI >30) impact your sex’s fertility?				0.267
Negatively impacts	300 (70.3%)	73 (63.5%)	227 (72.8%)	
Positively impacts	7 (1.6%)	3 (2.6%)	4 (1.3%)	
No impact	30 (7%)	9 (7.8%)	21 (6.7%)	
Unsure	90 (21.1%)	30 (26.1%)	60 (19.2%)	
How does being underweight (BMI <18.5) impact your sex’s fertility?				0.001
Negatively impacts	321 (75.2%)	71 (61.7%)^a^	250 (80.1%)^a^	
Positively impacts	2 (0.5%)	1 (0.9%)	1 (0.3%)	
No impact	28 (6.6%)	12 (10.4%)^a^	16 (5.1%)^a^	
Unsure	76 (17.8%)	31 (27%)^a^	45 (14.4%)^a^	
How does proper nutrition impact your sex’s fertility?				0.044
Negatively impacts	46 (10.8%)	5 (4.3%)^a^	41 (13.2%)^a^	
Positively impacts	333 (78.2%)	94 (81.7%)	239 (76.8%)	
No impact	18 (4.2%)	5 (4.3%)	13 (4.2%)	
Unsure	29 (6.8%)	11 (9.6%)	18 (5.8%)	
How does excessive alcohol impact your sex’s fertility?				0.019
Negatively impacts	346 (81%)	87 (75.7%)	259 (83%)	
Positively impacts	6 (1.4%)	5 (4.3%)^a^	1 (0.3%)^a^	
No impact	25 (5.9%)	7 (6.1%)	18 (5.8%)	
Unsure	50 (11.7%)	16 (13.9%)	34 (10.9%)	
How does marijuana impact your sex’s fertility?				0.030
Negatively impacts	235 (55%)	52 (45.2%)^a^	183 (58.7%)^a^	
Positively impacts	9 (2.1%)	5 (4.3%)	4 (1.3%)	
No impact	61 (14.3%)	21 (18.3%)	40 (12.8%)	
Unsure	122 (28.6%)	37 (32.2%)	85 (27.2%)	
How does smoking tobacco impact your sex’s fertility?				0.002
Negatively impacts	330 (77.3%)	75 (65.2%)^a^	255 (81.7%)^a^	
Positively impacts	3 (0.7%)	2 (1.7%)	1 (0.3%)	
No impact	31 (7.3%)	11 (9.6%)	20 (6.4%)	
Unsure	63 (14.8%)	27 (23.5%)^a^	36 (11.5%)^a^	
How does the influenza vaccine impact your sex’s fertility?				0.156
Negatively impacts	42 (9.8%)	12 (10.4%)	30 (9.6%)	
Positively impacts	22 (5.2%)	8 (7%)	14 (4.5%)	
No impact	193 (45.2%)	42 (36.5%)	151 (48.4%)	
Unsure	170 (39.8%)	53 (46.1%)	117 (37.5%)	
How does exogenous testosterone impact your sex’s fertility?				<0.001
Negatively impacts	115 (26.9%)	25 (21.7%)	90 (28.8%)	
Positively impacts	26 (6.1%)	21 (18.3%)^a^	5 (1.6%)^a^	
No impact	26 (6.1%)	8 (7%)	18 (5.8%)	
Unsure	260 (60.9%)	61 (53%)^a^	199 (63.8%)^a^	
How do anabolic steroids impact your sex’s fertility?				0.003
Negatively impacts	258 (60.4%)	78 (67.8%)	180 (57.7%)	
Positively impacts	7 (1.6%)	5 (4.3%)^a^	2 (0.6%)^a^	
No impact	19 (4.4%)	6 (5.2%)	13 (4.2%)	
Unsure	143 (33.5%)	26 (22.6%)^a^	117 (37.5%)^a^	
How does prior use of oral contraceptive pills impact a female’s fertility?				
Negatively impacts			115 (37.0%)	
Positively impacts			10 (3.2%)	
No impact			79 (25.4%)	
Unsure			107 (34.4%)	
How does prior use of long acting reversible contraceptives impact a female’s fertility?				
Negatively impacts			97 (31.4%)	
Positively impacts			8 (2.6%)	
No impact			90 (29.1%)	
Unsure			114 (36.9%)	
How does high blood pressure impact your sex’s fertility?				0.031
Negatively impacts	272 (63.8%)	63 (55.3%)^a^	209 (67%)^a^	
Positively impacts	6 (1.4%)	4 (3.5%)^a^	2 (0.6%)^a^	
No impact	26 (6.1%)	7 (6.1%)	19 (6.1%)	
Unsure	122 (28.6%)	40 (35.1%)	82 (26.3%)	
How does sexually transmitted disease impact your sex’s fertility?				0.116
Negatively impacts	277 (64.9%)	72 (62.6%)	205 (65.7%)	
Positively impacts	4 (0.9%)	3 (2.6%)	1 (0.3%)	
No impact	51 (11.9%)	11 (9.6%)	40 (12.8%)	
Unsure	95 (22.2%)	29 (25.2%)	66 (21.2%)	

Total participants (*n* = 427). Male (*n* = 115). Female (*n* = 312).

^a^ Indicates a significant difference in the responses between men and women.

**FIG. 1.**
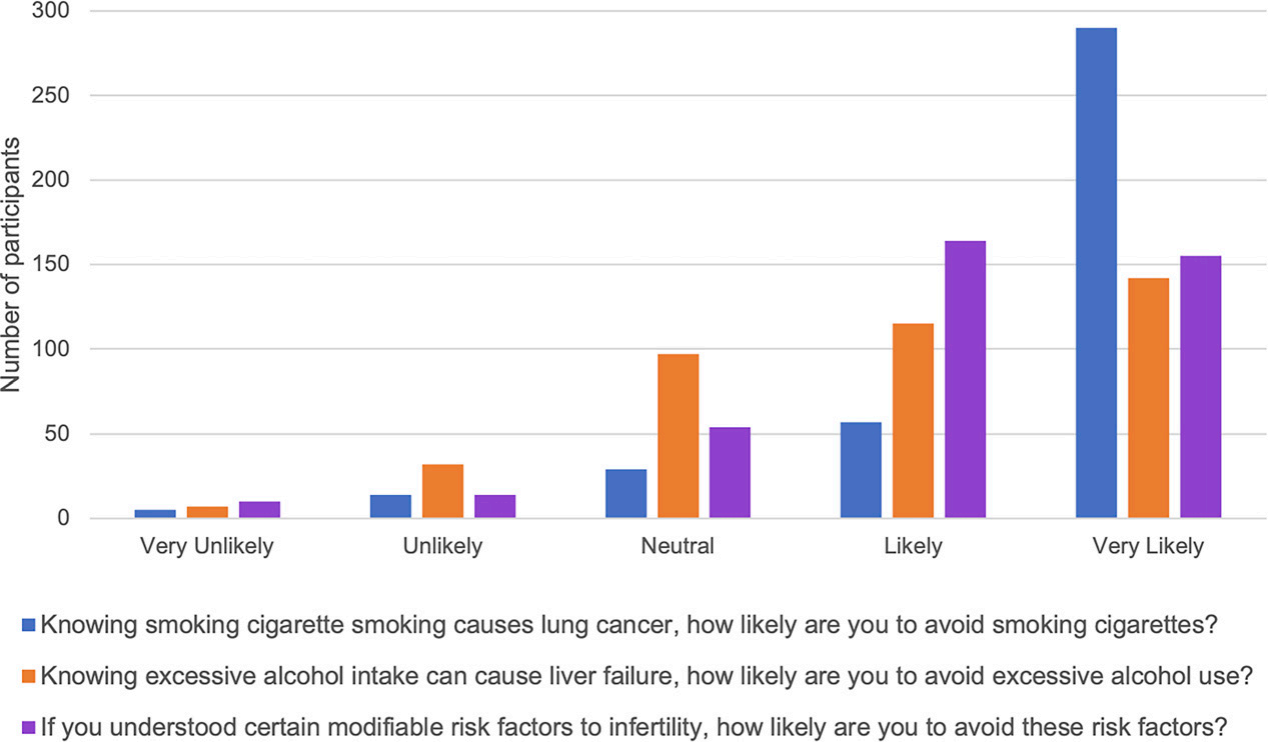
How likely are young adults to change their behavior? Comparison of how likely participants are to change their behavior knowing the negative health consequences of smoking tobacco, excessive alcohol use, or modifiable risk factors for infertility. Total participants *n* = 427.

Participants ranked statements on a Likert scale to estimate how likely they were to change a behavior knowing its negative health consequences (Fig. 1). With a rating of 1 being not likely and 5 being very likely, participants were more likely to avoid risk factors that negatively impact fertility (4.11 ± 0.942). Participants were significantly more likely to avoid smoking to prevent lung cancer than to avoid alcohol to prevent liver disease or to avoid risk factors for infertility (*p* < 0.001). However, participants were significantly more likely to avoid certain modifiable risk factors to prevent infertility than they were to avoid excessive alcohol to prevent liver disease (*p* = 0.008). There was no difference between males and females in how likely they were to avoid behaviors to prevent negative health outcomes (*p* > 0.05).

About 47% of females and 63% of males selected age as the leading nonmodifiable risk factor for fertility. Eight percent of total participants selected ethnicity, 10% chose abnormal anatomy, and 31% selected genetic predisposition as the leading nonmodifiable risk factor for infertility.

### Self-reflection on understanding and importance of fertility

Most of the participants felt that they have a moderate understanding of their own fertility, though nearly half of respondents reported they wish they knew more about fertility. When the importance of fertility was rated on a Likert scale, 1 being not at all important and 5 being extremely important, the mean was 3.57 ± 1.177 (Table 2). The largest percentage of male participants, 34.4%, reported learning the most about fertility in high school health courses, followed by 26.5% from college courses, 13.7% from family and friends, 9.8% from a physician, 9.8% from social media, and 5.9% from an unspecified source. The largest percentage of females reported learning the most about fertility from social media (22.8%). Females reported learning about fertility from high school sex education (20.5%), family and friends (17.8%), a physician (16.8%), college courses (14.4%), or an unspecified source (7.9%). Significantly more females reported learning about fertility from social media (*p* < 0.05), while significantly more males reported learning the most about fertility from high school sex education courses (*p* < 0.05). However, 50% of males and 56.4% of females reported they think the best place to learn about their fertility is from a physician.

**Table 2.** Attitudes Regarding Fertility Awareness and Importance

	Male (*n* = 115)	Female (*n* = 312)
How well do you understand what affects your own sex’s fertility?		
Poor	8%	7%
Minimal	32%	25%
Moderate	39%	45%
Good	15%	18%
Strong	6%	5%
How important is fertility to you—the ability to have your own children?		
not at all important	6%	8%
slightly important	12%	8%
moderately important	25%	28%
very important	40%	28%
extremely important	17%	28%
How much do you wish you knew about your fertility?		
A little	21%	14%
A moderate amount	60%	45%
A lot	19%	41%

Total participants *n* = 427. Total males *n* = 115. Total females *n* = 312.

The number of responses to each question may differ from the total number of participants because some participants may have chosen to forego answering specific questions.

## Discussion

This study demonstrates specific knowledge gaps in university students’ understanding of modifiable risk factors for infertility. Through asking how different lifestyle choices affect fertility, it is clear that young adults need education on how the influenza vaccine, reversible contraceptive methods, and exogenous testosterone impact fertility. The ability to have children is important to most of the respondents, and they are willing to make changes to their behaviors to prevent infertility. However, they must first understand what negatively impacts their fertility before they can make any changes. Having accurate sources of information to develop this understanding is critical.

### Understanding of modifiable risk factors

Undergraduate students have some understanding of risk factors for infertility. It is reassuring that the majority of females and males in this study determined stress, obesity, and being underweight as negatively impacting fertility. This is in agreement with the CDC and ASRM.^14,27^ While recognizing that current data on the most beneficial diet for fertility is uncertain, ASRM does recognize that a healthy lifestyle improves overall health and would contribute to optimizing natural fertility, of which the majority of females and males in this study had a clear understanding.

One significant misunderstanding among participants was whether the influenza vaccine impacts fertility. The influenza vaccine is recommended for individuals without contraindications, and the CDC and American College of Obstetricians and Gynecologists both recommend the vaccine specifically for women trying to conceive or who are pregnant.^29,30^ The vaccine has not been shown to have any negative effects on fertility.^31^ In a time of rising vaccine hesitancy, it is important to minimize the misconceptions surrounding vaccines.^32^ There was also widespread misinformation and misconceptions between the COVID-19 vaccine and infertility.^33^ Considering that 46% of males and 38% of females in this study were unsure on how the influenza vaccine impacts fertility, there is a need to dispel any misunderstandings that the influenza and COVID-19 vaccine can negatively impact fertility.

Because of the prevalence of STDs, this study also identifies that additional education on how STDs may impact female fertility is warranted. Though most females in this study understood STDs negatively impact fertility, the second most common answer was “unsure” (21.2%). The number of chlamydia and gonorrhea cases have increased nationally from 2020 to 2021.^34^ Both primary care physicians and Ob/Gyn physicians should counsel young adults on safe sex practices to prevent STDs and provide frequent screening for gonorrhea and chlamydia in sexually active women under the age of 25, as suggested by the United States Preventive Services Task Force guidelines.^35^

In this study, most males and females determined excessive alcohol use, smoking tobacco, and marijuana use can negatively impact fertility. The CDC identifies that smoking, excessive alcohol intake, and drug use may increase the risk of infertility for both sexes.^27^ The ASRM similarly recognizes smoking as a negative contributing factor to female fertility. However, though studies show sperm abnormalities in men who smoke, there is no data to definitively claim tobacco smoking decreases male fertility.^14^ While a minimal or moderate amount of alcohol can variably affect fertility, one study showed an increasing amount of alcohol is associated with decreased conception for women. Men who consume alcohol in excessive amounts have been shown to have increased follicle-stimulating hormone and luteinizing hormone levels along with decreased testosterone levels and sperm quality.^36^ A recent review highlights that marijuana can cause ovulatory dysfunction and menstrual cycle irregularities and can decrease sperm motility and quantity.^37^ However, in 2018, data from the National Survey of Family Growth showed marijuana use by men or women does not significantly change the time to pregnancy.^38^ Ultimately, the ASRM discourages the use of marijuana for men and women desiring pregnancy.^14^

It is important to consider the possibility that participants selected the answer choice “negatively impacts fertility” based on the general association between smoking tobacco, marijuana use, and excessive alcohol consumption and negative health outcomes rather than determining their impact on fertility specifically. These behaviors are prevalent in college-aged individuals and the use of marijuana by college students has increased due to its recent widespread legalization.^39^ It is critical to continue investigating how, or to what extent, marijuana affects fertility for men and women. While there is uncertainty in the literature about how marijuana affects fertility, the American Urologic Association recommends providers discuss the possible complications of marijuana in reproductive-aged individuals.^14,27,40^

Another misconception was identified regarding testosterone use. Only 22% of males reported that exogenous testosterone negatively impacts male fertility, in contrast to the 67.8% of males who identified anabolic steroids as having a negative impact on fertility. Testosterone and anabolic steroids have an established detrimental impact on male fertility due to their impact on intratesticular testosterone production through hypogonadotropic hypogonadism.^41^ This decrease in function of the hypothalamic-pituitary-gonadal axis has been shown to be reversible in men, but there is little data to suggest the long-term effects in women.^42,43^ Because fertility is time-sensitive, most significantly for females, this temporary reduction in fertility may be highly unfavorable for an individual’s family planning goals. One study showed a four-fold increase in men using testosterone replacement between 2003 and 2013.^44^ With an increasing number of men using testosterone products, adequate patient counseling must take place to ensure patients are making educated decisions on the risks and benefits of testosterone therapy.

Interestingly, when asked about prior use of reversible forms of birth control, many women had misconceptions that they negatively impact fertility or are unsure how they impact fertility. Regarding oral contraceptive pills, 37% of women believe they negatively impact fertility and 34% are unsure. Thirty-one percent of females believe long-acting reversible contraceptives negatively impact their fertility, whereas 37% of females are unsure. The numbers from this study are similar to that of a 2022 systematic review and meta-analysis which found that 37% of women believed LARCs can cause infertility and a survey in Denmark showing 45.5% of current oral contraceptive (OCP) users believed getting pregnant would be more difficult following discontinuation of use.^45,46^ The CDC and ASRM do not support that OCPs or LARCs are harmful to future fertility. A 2018 systematic review concluded that there is no long-term negative impact on fertility after the discontinuation of oral contraceptives.^47^ There should be more discussion between providers and their patients regarding the risks and benefits of contraceptive options, including a discussion on family planning. Pediatricians, primary care providers, and gynecologists all have the capability of having these conversations with patients to dispel the myths surrounding the impact of reversible forms of contraception on fertility.

### Strengths and weaknesses

This study was successful in understanding how well college students understand modifiable risk factors to their fertility, and how much they value their fertility and elicits further discussions on how to educate young adults on their fertility. Many surveys on fertility knowledge include only women or only men participants; however, this study was designed to include both genders. The survey was selective in including only UNR students; however, this reduces any confounding factor that level of education creates. The number of responses collected was well over the intended minimum of 100.

There are limitations to this study. During survey validation, the pilot survey sample size was small and did not contain survey experts. The respondents were largely women and between the ages of 18 and 20. Women have previously been shown to have a higher interest in fertility than men and have a better understanding of their fertility compared with men.^18^ This may explain the disproportionate number of women who participated in the study and a possible positive inflation of correct responses overall. When recruiting participants, an effort was made to attend a variety of college courses. However, most of the classes that the researchers were able to recruit participants for were entry-level courses which may have contributed to the large number of 18- to 20-year-old individuals. The courses were more heavily attended by students with science majors. Those with a science major may have been more knowledgeable on fertility. The demographics outside of age, sex, and gender were not analyzed. Other studies investigating young adults’ knowledge of fertility showed that there were significantly different responses when stratifying participants by race or socioeconomic status. Further evaluation of how different demographics understand modifiable risk factors to fertility would be important in tailoring education toward groups with a poorer understanding of these behaviors. Lastly, there are limitations with a web-based survey. Students may have used online resources at the time of the survey to guide their answer choices.

## Conclusion

Receiving a diagnosis of infertility can be devastating, comparable to that of a cancer diagnosis.^48^ Despite the great consequences of infertility, our study shows young people have a poor understanding or misconceptions of factors that impact infertility. However, study participants want to learn more about their fertility. Furthermore, they indicated that they were likely to make changes based on new knowledge of their fertility. Young adults in this study are more likely to avoid behaviors that would put them at risk for infertility than they would avoid excessive alcohol use to prevent liver disease. In order to avoid risk factors for infertility, young adults must be educated on what those risk factors are. Our study contributes to existing literature that emphasizes the need for greater fertility education targeting the younger population.^17,20^

Fertility counseling and family planning discussions are an important component of preventative health care. Though most participants felt a physician would be the ideal source of information, it does not align with how they are currently informed. With the largest percentage of women learning about fertility from social media, there is a significant concern that their information is not evidence-based. A multidisciplinary approach between various physicians and Ob/Gyn physicians is important for delivering preventative health education to young adults. This information could help young individuals avoid the financial, emotional, and physical stress of infertility treatments. It is currently unknown how common these conversations on fertility or family planning are in primary health clinics. Further work must be done to understand how this knowledge gap can be most effectively addressed so that young adults can potentially avoid the detrimental consequences of infertility.

## Supplementary Material

Supplementary Appendix SA1

## Abbreviations Used

ART: assisted reproductive technology
ASRM: American Society of Reproductive Medicine
CDC: Centers for Disease Control and Prevention
LARCs: long acting reversible contraceptives
OCPs: oral contraceptive pills
STDs: sexually transmitted diseases
UNR: University of Nevada, Reno
