# Supplementary material for: Young Adults’ Understanding of Modifiable Risk Factors of Infertility
Source: Womens Health Rep (New Rochelle). 2024 Oct 22;5(1):815–24. doi: 10.1089/whr.2024.0058 (PMC11496944; doi:10.1089/whr.2024.0058)
Supplement: Supplementary Appendix SA1 [file whr.2024.0058_supp_datas1.docx]

**Behavioral Factors that Impact Fertility**

**Consent Form**
You are being invited to participate in a research study. Before you agree to be in the study, read this form carefully. Participation is voluntary; there are no negative consequences by choosing not to participate. If you agree now but change your mind, you may quit the study at any time while taking the survey.

**Study Purpose:** The aim of this study is to identify if young adults have deficits in knowledge regarding what factors may positively or negatively impact fertility.

**Your Participation:** The survey will take about 5 minutes. There is no personally identifying information requested as part of this study other than your age, gender, assigned birth sex, and if you are enrolled at the University of Nevada, Reno. To be included in this study, participants must be between age 18-26, who are enrolled in at least one course at the University of Nevada, Reno. This study is considered to be minimal harm. 

The researchers and the University of Nevada, Reno School of Medicine will treat the survey data collected with professional standards of confidentiality and protect it to the extent allowed by law.

If you have questions at any point during your participation, you may contact one of the researchers: Lauren Lim- llim@med.unr.edu or Mimi Hoppe- meredithh@med.unr.edu.

[**Click here for the full consent form**](https://docs.google.com/document/d/1c7U1B4CJPd0HRA4hkgO_pUex6CGQUteU/edit?usp=sharing&ouid=114851671170177253757&rtpof=true&sd=true)

**Confirmation of Your Consent By clicking “yes” below, you are consenting to participate in this study and acknowledge the following: You understand the risks and benefits to this study, you understand you can stop the survey at any time without negative repercussions, and you are 18 years of age.**

1. What is your assigned birth sex?

Male

Female

1. What is your gender?

Male

Female

Non-binary/third gender

Prefer not to say

1. Do you take at least one class at the University of Nevada, Reno? (can be undergraduate or graduate programs)

Yes

No

1. What is your age?

18-20

21-23

24-26

>26

The following questions are to assess your understanding of how modifiable behaviors impact fertility. **Fertility, for the purpose of this study, is the likelihood of establishing a pregnancy in 12 months of regular, unprotected sexual intercourse.** Fertility may be impacted by male and female anatomy, male sperm count and motility, female egg count, uterine environment, and other factors.

Questions ask you to identify how certain factors influence fertility **based on your birth sex**. If responding to questions regarding your birth sex is triggering, you are welcome to discontinue the survey with no negative consequence. The answer choices are negatively impacts, positively impacts, no impact, or unsure. Please answer the questions to the best of your ability without using outside resources.

1. How does stress impact your sex's fertility?

Negatively impacts

Positively impacts

No impact

Unsure

1. How does obesity (BMI >30) impact your sex's fertility?

Negatively impacts

Positively impacts

No impact

Unsure

1. How does being underweight (BMI<18.5) impact your sex's fertility?

Negatively impacts

Positively impacts

No impact

Unsure

1. How does proper nutrition impact your sex's fertility?

Negatively impacts

Positively impacts

No impact

Unsure

1. How does excessive alcohol impact your sex's fertility?

Negatively impacts

Positively impacts

No impact

Unsure

1. How does marijuana impact your sex's fertility?

Negatively impacts

Positively impacts

No impact

Unsure

1. How does smoking tobacco impact your sex's fertility?

Negatively impacts

Positively impacts

No impact

Unsure

1. How does the influenza vaccine impact your sex's fertility?

Negatively impacts

Positively impacts

No impact

Unsure

1. How does exogenous testosterone impact your sex's fertility?

Negatively impacts

Positively impacts

No impact

Unsure

1. How does anabolic steroids impact your sex's fertility?

Negatively impacts

Positively impacts

No impact

Unsure

1. How does prior use of oral contraceptive pills impact a female's fertility?

Negatively impacts

Positively impacts

No impact

Unsure

Birth sex not female

1. How does prior use of long acting reversible contraceptives impact a female's fertility (IUDs, nexplanon)?

Negatively impacts

Positively impacts

No impact

Unsure

Birth sex not female

1. How does high blood pressure impact your sex's fertility?

Negatively impacts

Positively impacts

No impact

Unsure

1. How does sexually transmitted disease impact your sex's fertility?

Negatively impacts

Positively impacts

No impact

Unsure

1. What is the leading NON-modifiable risk factor for female infertility?

Age

Ethnic Group

Genetic Predisposition

Abnormal anatomy

1. How well do you understand what affects your own sex's fertility?

Poor Understanding

Minimal Understanding

Moderate Understanding

Good Understanding

Strong Understanding

1. How important is fertility to you- the ability to have your own children?

Not at all important

Slightly important

Moderately important

Very important

Extremely important

Rank the following statements:

1. Knowing smoking cigarette smoking causes lung cancer, how likely are you to avoid smoking cigarettes?

Very Unlikely, Unlikely, Neutral, Likely, Very Likely

1. Knowing excessive alcohol intake can cause liver failure, how likely are you to avoid excessive alcohol use?

Very Unlikely, Unlikely, Neutral, Likely, Very Likely

1. If you understood certain modifiable risk factors to infertility, how likely are you to avoid these risk factors?

Very Unlikely, Unlikely, Neutral, Likely, Very Likely

1. How much more do you wish you knew about your fertility?

A little

A moderate amount

A lot

1. Where have you learned the most about your fertility

Physician

High School Sex Education

College Courses

Social Media

Family and Friends

Other

1. Where do you think would be the best place to learn about your fertility?

High School Sex Education

Social Media

Physician

College Courses

Other

Thank you for taking the survey. If you are interested in learning more about factors that affect infertility, please visit these resources:

[**Centers for Disease Control: Infertility FAQs**](https://www.cdc.gov/reproductivehealth/infertility/index.htm)

[**American Society for Reproductive Medicine: Protecting Your Fertility**](https://www.asrm.org/topics/topics-index/?filterbycategoryid=109)
